# Karnofsky Performance Status as A Predictive Factor for Cancer-Related Fatigue Treatment with Astragalus Polysaccharides (PG2) Injection—A Double Blind, Multi-Center, Randomized Phase IV Study

**DOI:** 10.3390/cancers11020128

**Published:** 2019-01-22

**Authors:** Cheng-Hsu Wang, Cheng-Yao Lin, Jen-Shi Chen, Ching-Liang Ho, Kun-Ming Rau, Jo-Ting Tsai, Cheng-Shyong Chang, Su-Peng Yeh, Chieh-Fang Cheng, Yuen-Liang Lai

**Affiliations:** 1 Division of Hematology/Oncology, Department of Internal Medicine, Chang Gung Memorial Hospital, Chang Gung University, College of Medicine, Keelung 204, Taiwan; wwww9208@ms19.hinet.net; 2 Chi Mei Medical Center, Liouying, Tainan City 736, Taiwan; cylin0615@yahoo.com.tw; 3 Department of Hematology/Oncology, Linkou Chang Gung Memorial Hospital, Taoyuan 333, Taiwan; js1101@cgmh.org.tw; 4 College of Medicine, Chang Gung University, Taoyuan 333, Taiwan; 5 Tri-Service General Hospital, Taipei 114, Taiwan; hochingliang@yahoo.com.tw; 6 Hematology-Oncology Division, Kaohsiung Chang Gung Memorial Hospital, Kaohsiung 833, Taiwan; liu07822@ms57.hinet.net; 7 Hematology-Oncology Department, E-Da Cancer Hospital, Kaohsiung 824, Taiwan; 8 Collage of Medicine, I-Shou University, Kaohsiung 824, Taiwan; 9 Department of Radiology, School of Medicine, College of Medicine, Taipei Medical University, Taipei 11031, Taiwan; kitty4024@gmail.com; 10 Department of Radiation Oncology, Shuang Ho Hospital, Taipei Medical University, New Taipei City 23561, Taiwan; 11 Changhua Christian Hospital, Changhua County 500, Taiwan; 15120@cch.org.tw; 12 China Medical University Hospital, Taichung City 40447, Taiwan; supengyeh@gmail.com; 13 Phytohealth Co., Taipei 105, Taiwan; cfcheng@phytohealth.com.tw; 14 MacKay Memorial Hospital, New Taipei City 25160, Taiwan; 15 Mackay Medical College, New Taipei City 25245, Taiwan

**Keywords:** astragalus polysaccharides, fatigue, cancer, palliative care, KPS

## Abstract

Fatigue is a common and debilitating symptom in patients with advanced cancer, resulting in poor quality of life and reduced treatment efficacy. Phytotherapeutic agents have shown potential effects to relieve cancer-related fatigue in these patients. The aim of this study was to evaluate the efficacy and safety of Astragalus Polysaccharides injection and identify predictive factors associated with this treatment. Patients with advanced cancer receiving palliative care with moderate to severe cancer-related fatigue were enrolled in this study for two treatment cycles. Fatigue improvement response rates were analyzed as the primary endpoint at the end of the first cycle to determine treatment efficacy. The drug safety profile was evaluated by the reporting of adverse events. Three hundred and ten patients were enrolled in this study and 214 patients were included ITT population. Improvement in fatigue scores by at least 10% was observed in greater than 65% of subjects after one treatment cycle compared to scores at baseline. Patients with higher Karnofsky Performance Status (KPS) responded better to the Astragalus Polysaccharides injection. Drug-related adverse event rates were less than 9%. This study identified KPS as a promising predictive factor for the therapeutic efficacy of Astragalus Polysaccharides injection.

## 1. Introduction

Fatigue is reported in 60% to 90% of patients with advanced cancer [1,2]. Cancer-related fatigue (CRF, also known as neoplastic related fatigue) is characterized by feeling tired, weak, exhausted, lethargic, and lacking energy, as well as being unable to concentrate or feel motivated [3]. Due to insomnia or overexertion, CRF is considered more severe and disabling than fatigue due to other causes based on patient reports [4]. The intensity and duration of fatigue is significantly higher in breast cancer patients and survivors than in control patients with benign breast problems, and fatigue results in the deterioration of quality of life in cancer patients and survivors [5,6].

Fatigue is one of the chief complaints of cancer patients seeking emergency care, and patients, with severe fatigue are more likely to be unstable and unable to return home after emergency care [7]. A long-term study also demonstrated that 34% of cancer patients reported significant fatigue 5–10 years after diagnosis [8]. Furthermore, neoplastic (malignant)-related fatigue was assigned R53.0 as a billable/specific ICD-10-CM diagnostic code. Despite reported severity, CRF is often neglected.

Cancer-related fatigue is a multidimensional syndrome caused by a number of physical and psychosocial mechanisms, including tumor by-products, cytokine-induced cachexia, muscle loss, or deconditioning [9,10]. Limited specific pharmaceutical interventions to ameliorate cancer-related fatigue are available [11]. Inflammation is an important factor that may be involved in the etiology of CRF [12]. Interestingly, Astragalus Polysaccharides were reported to have antioxidant and anti-inflammatory effects [13]. Therefore, Astragalus Polysaccharides (PG2) injection is approved as a type of pharmacotherapy for the treatment of cancer-related fatigue in advanced cancer patients in Taiwan.

PG2 injection is prepared from a sterile powder of polysaccharides isolated from Huang qi. Huang qi, the root of *Astragalus membranaceus var. mongholicus* (AM), is one of the most common Chinese herbs used to treat patients suffering from various conditions collectively described as “Chi-shiu” (deficiency of vital energy) and anemia (deficiency of blood). Previous pre-clinical and clinical results showed that PG2 treatment may provide clinical benefits to advanced cancer patients. Potential clinical benefits include reduced fatigue, improved quality of life, enhanced immunity, and stabilization of weight loss [14,15]. A meta-analysis for non-small cell lung cancer patients receiving platinum-based chemotherapy concluded that Astragalus-based Chinese herbal medicine may increase the effectiveness of platinum-based chemotherapy by improving survival, tumor response, and performance status (Karnofsky Performance Status) [16].

To further elucidate the effects of PG2 injection, this large-scale phase IV study aimed to determine the efficacy of PG2 injection in relieving fatigue. In addition, we evaluated the safety profile of PG2 injection in advanced cancer patients. We further identified Karnofsky Performance Status (KPS) as a promising predictive factor for therapeutic effects of PG2.

## 2. Results

### 2.1. Patient Characteristics

**Figure 1:**
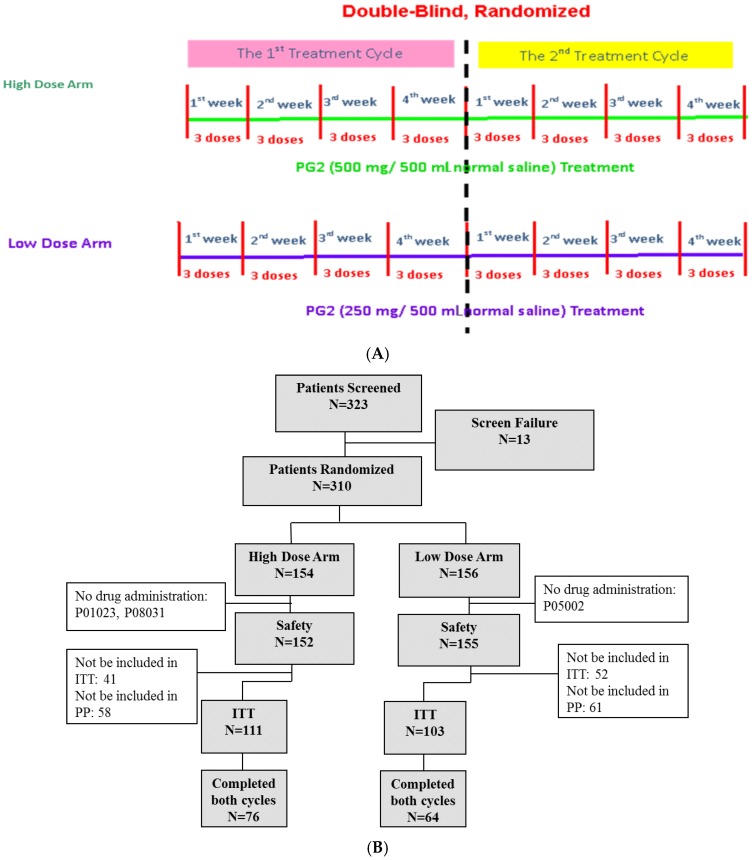
Study flow chart and patient flow diagram. (**A**) Patients were randomly divided into high dose (500 mg) and low dose (250 mg) groups. Each patient was expected to receive two cycles of treatment. Each cycle lasted 4 weeks, and patients received three doses of PG2 injection per week. (**B**) Three hundred and twenty-three patients were screened and 310 were randomly divided into two groups. Three hundred and seven patients were enrolled in the safety population, and 214 were included in the intention-to-treat population.

Three hundred and twenty-three subjects were screened for eligibility, and 310 subjects were enrolled in this study. Of the enrolled subjects, 154 subjects were randomized and assigned to receive the high dose (500 mg) and 156 subjects were randomized to receive the low (250 mg) dose of PG2. Three subjects discontinued participation in the study before treatment with the study drug due to disease progression, death, or decreased fatigue score after enrolment (1%). A total of 214 subjects (69.0%) completed the baseline assessment and primary endpoint evaluation and were included in the intention-to-treat population. One hundred and forty subjects (45.2%) completed the study (Figure 1B).

Patients were randomly assigned to high dose (500 mg) or low dose (250 mg) treatment groups. All baseline demographic characteristics, including age, sex, cancer type, and disease status, were comparable in both groups (Table 1). The average age of patients was 62.2 years old in the high dose group and 62.86 years old in the low dose group. Participants were diagnosed with various types of cancer, including lung, breast, head and neck, pancreas, stomach, colorectal, liver, and others. In both groups, lung cancer, colorectal cancer, and breast cancer were the most frequent cancer types and accounted for more than 40% of the patients in the study (Supplementary Table S2). The average KPS was 64.5 in the high dose group and 66.65 in the low dose group, and the average Brief Fatigue Inventory-Taiwanese version score was 6.8 in the high dose group and 6.76 in the low dose group (Table 1).

### 2.2. Cancer-Related Fatigue Assessment

**Figure 2:**
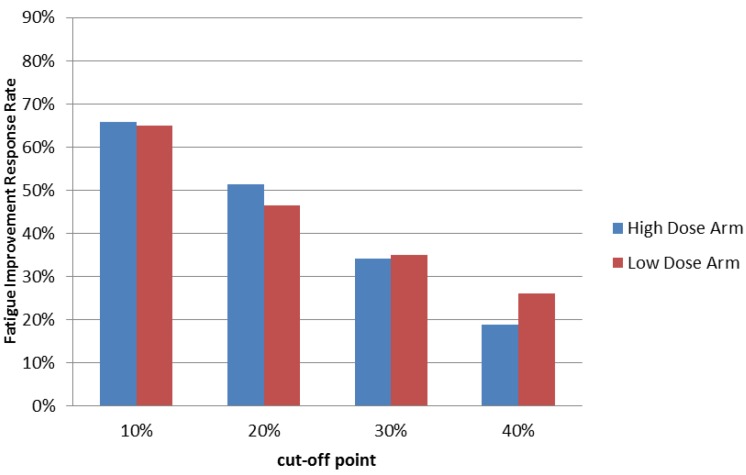
Summary of Fatigue Improvement Response Rate at the end of treatment cycle 1. Patients who received high or low dose PG2 treatments were evaluated using the Brief Fatigue Inventory-Taiwanese version at the first visit (baseline) and once each following week. Fatigue improvement response rates using different cut off scores were calculated at the end of treatment cycle 1.

Our previous study demonstrated that PG2 injection can improve fatigue in more than 60% treated cancer patients. Consistent with our previous study, administration of PG2 injection resulted in a greater than 65% fatigue-improvement response rate with 10% improvement or more in patients after one cycle of treatment. These results were similar in both treatment groups (Figure 2 & Table 2). Effects were observed as early as the third visit of week 1 in both high and low dose groups (*p* < 0.0001 in both groups), and the effect lasted throughout the entire study (Supplementary Table S3).

Furthermore, although a 10% improvement in the Brief Fatigue Inventory-Taiwanese version score was the threshold for being defined as a responder, 78% (57/73) of the 66% (73/111) of responders in the high dose group reported greater than 20% improvement, while 72% (48/67) of the 65% (67/103) of responders in the low dose group reported greater than 20% improvement at the end of cycle 1. Over half of the responders reported greater than 30% improvement in both groups. Interestingly, we found that 29% (21/73) of responders in the high dose group and 40% (27/67) of responders in the low dose group reported greater than 40% improvement at the end of cycle 1 (Figure 2 & Table 2).

Since cancer patients usually undergo other concurrent treatments, we further analyzed a subpopulation without chemotherapy, radiation therapy, or steroid treatment. The majority of patients in this category also showed a response to PG2 in terms of BFI score improvement (Supplementary Figure S1).

**Figure 3:**
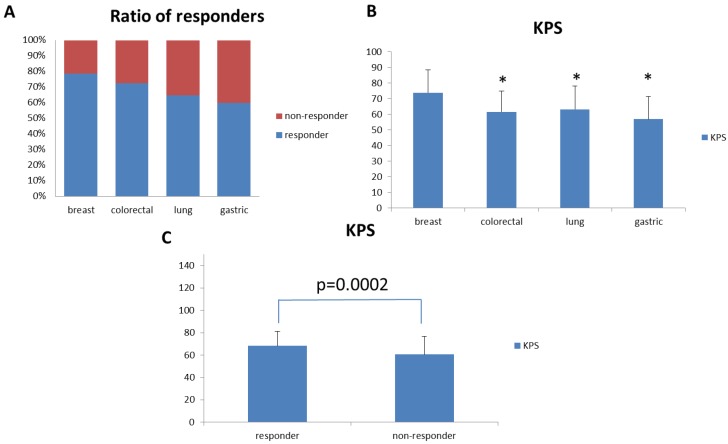
Fatigue Improvement Response Rate and KPS for patients with different cancer types. (**A**) Breast, colon, lung, and gastric cancer patients were selected for analysis. Fatigue improvement response rates for these patients were analyzed and compared. (**B**) KPS for breast, colon, lung, and gastric cancer patients were analyzed and compared. (**C**) KPS for responders and non-responders in the overall patient population. (* *p* < 0.01 versus breast cancer patients).

Since we enrolled patients with different types of cancer, we sought to analyze the effect of PG2 injection on cancer type-specific fatigue improvement. We evaluated cancer types with twenty or more patients enrolled in this study. Therefore, we analyzed the effect of PG2 injection on fatigue improvement in lung cancer (34 patients), colorectal cancer (29 patients), breast cancer (28 patients), and gastric cancer (20 patients) patients. Among these cancer patients, breast cancer patients showed the best response to PG2 injection (Figure 3A). Interestingly, breast cancer patients also showed significantly higher average baseline KPS when compared to colorectal, lung, and gastric cancer patients (Figure 3B).

Based on this observation, we further analyzed the average baseline KPS between responders and non-responders to PG2 injection in the intention-to-treat population. The average baseline KPS was significantly higher in responders than in non-responders (Figure 3C). We further analyzed the relationship between baseline KPS score and responder status. There was a significant relationship between baseline KPS score and responder status, as determined by univariate analysis. Responder subjects had higher baseline KPS scores than non-responder subjects (*p* < 0.0001). Multivariate analysis showed that the baseline KPS score (*p* < 0.0001) correlated with responder status after adjusting for other variables. The odds of being a responder with a baseline KPS score of 30–50 was 0.253 times lower than for subjects with a +60 baseline KPS score (Table 3). We also found that the baseline KPS score correlated with the responder status in both the high and low dose groups (Supplementary Tables S5 and S6).

### 2.3. Toxicity Evaluation

A total of 1750 adverse events were reported in this study. However, only five symptoms were classified as treatment-related adverse events with over 2% incidence (Table 4). In summary, more than 90% of reported adverse events were unlikely to be related or not related to PG2 treatment. More than 80% of reported adverse events were classified as “mild (grade 1)” or “moderate (grade 2)” (Supplementary Table S4).

## 3. Discussion

Cancer-related fatigue is one of the most common and difficult to treat symptoms during cancer treatment, and fatigue and depression might coexist and overlap considerably in cancer patients [17,18,19]. The prevalence of diagnosable cancer-related fatigue is 49.8% according to ICD-10 criteria in a recent study [20]. The prevalence might be even higher when judged by other criteria. In our study, 81.8% of patients (intention-to-treat population, Brief Fatigue Inventory-Taiwanese version ≥4) were diagnosed with cancer-related fatigue by the ICD-10 criteria. The purpose of this study was not to directly compare the two doses used in this study but to demonstrate the therapeutic effect of PG2 injection in CRF. In this study, we demonstrated that PG2 injection could be an effective and safe treatment for relieving fatigue in advanced cancer patients. We further confirmed the effect of PG2 injection in treating cancer-related fatigue in this large-scale study in patients with multiple cancer types.

Although cancer-related fatigue is highly prevalent in cancer patients and is classified in the World Health Organization’s ICD-10-CM, options to relieve cancer-related fatigue are limited. Current suggestions for cancer-related fatigue include non-pharmacologic interventions and pharmaceutic treatments [21]. Exercise is the most widely studied and approved non-pharmacologic intervention for cancer-related fatigue. Cognitive-behavioral therapy, sleep hygiene, and nutritional supplementation are also accepted non-pharmacologic interventions, depending on patient status. For patients with moderate to severe cancer-related fatigue, drug treatment may be necessary. Methylphenidate is a CNS stimulant and can be used off-label for treatment of cancer-related fatigue. Methylprednisolone and other steroids may also relieve cancer-related fatigue due to their anti-inflammatory activities. Both high and low doses of Astragalus polysaccharides injection have been demonstrated to relieve cancer-related fatigue in previous studies and in our current study [14,22].

Fatigue is a common symptom experienced by cancer patients. However, it was reported that the prevalence and severity of fatigue could be different among patients with different cancer types [23]. Due to different treatment protocols and stages of disease, it is difficult to define the correlation between cancer types and fatigue experiences. In this study, we observed that PG2 injection improved fatigue response rates by over 75% in breast cancer patients, but only 60% in gastric cancer patients.

However, we also observed that the average KPS score was significantly higher in breast cancer patients than in patients with the three other major cancers in this study. Since KPS reflects the overall healthiness of cancer patients, patients with higher KPS may respond better to PG2 injection treatment. This may explain the observation that patients with different types of cancer responded differently to PG2 injection treatment. Indeed, when we tested this hypothesis in our intention-to-treat population, we observed that the baseline average KPS of PG2 responders was significantly higher than that of non-responders. Furthermore, we demonstrated that KPS is a predictive factor for CRF patients treated with PG2.

KPS was invented by Dr. Karnofsky. The purpose of KPS is to evaluate the performance of cancer patients [24]. Indeed, higher fatigue scores were associated with lower KPS scores [10]. Our study is consistent with these previous reports. Furthermore, we identified KPS as a promising predictive factor to evaluate the therapeutic effect of PG2.

Cancer-related fatigue is a systemic disease, potentially resulting from many underlying mechanisms. We demonstrated that PG2 injection could effectively relieve fatigue in advanced cancer patients. Evaluation of the potential role of the cytokine profile is also under investigation. Studies in breast cancer survivors demonstrated that increased NF-κB in leukocytes may be a signal of persistent fatigue [25]. Cytokine dysregulation is also a known factor in the development of cancer-related fatigue [26]. TNFα is an important cytokine regulator of inflammatory and immune responses and has implications as an inducer of cancer-related fatigue [27]. Interestingly, Astragalus polysaccharide was reported to regulate the inflammatory response by reducing TNFα secretion in Caco2 cells [28]. In contrast, high dose Astragalus polysaccharides might increase the Th17 cell population, which is known to promote the production and secretion of proinflammatory cytokines [29]. Further studies are required to clarify the mechanism of Astragalus polysaccharide in relieving cancer-related fatigue.

To the best of our knowledge, this is the first large-scale randomized clinical trial to study the efficacy and safety of pharmacological treatment for cancer-related fatigue with over 300 advanced cancer patients enrolled. This study not only highlights the importance of investigating fatigue experienced by cancer patients, but also provides evidence for an effective treatment for fatigue.

As with other trials that have evaluated advanced cancer patients, the number of adverse events was high, and only a limited number of patients were able to complete both cycles of treatment due to disease progression. Moreover, due to ethical considerations for this post-market phase IV trial, a placebo arm was not included in this study. However, the superior effect of PG2 injection over placebo control in improving cancer-related fatigue was demonstrated in our earlier study [14].

## 4. Materials and Methods

### 4.1. Investigational Drug

The investigational drug PG2 injection (PhytoHealth Corp., Taiwan) was extracted from Astragalus membranaceus as previously described [14]. PG2 is characterized as a mixture of polysaccharides prepared as a lyophilized powder prior to use. For administration, each vial of 500 mg or 250 mg PG2 was reconstituted in 10 mL normal saline. The solution was then injected into normal saline (490 mL) and mixed well for intravenous infusion at a rate of 150 to 200 mL/h.

### 4.2. Patients

We proposed to recruit at least 200 evaluable inpatients and outpatients with advanced cancer who were undergoing standard palliative care at 9 hospitals in Taiwan. The inclusion criteria were as follows: (1) Patients with locally advanced, metastatic, or inoperable advanced cancer. (2) Patients under palliative care in a hospice setting with no other curative options available. (3) Patients with Brief Fatigue Inventory-Taiwanese version score ≥4 at screening. Exclusion criteria were as follows: (1) Patients with uncontrolled systemic disease, such as active infection, severe heart disease, uncontrollable hypertension, or diabetes mellitus. (2) Patients who had taken central nervous system stimulators, such as methylphenidate, within 30 days before screening. (3) Patients with KPS scores of less than 30 at screening. This clinical trial was reviewed and approved by the Institutional Review Board of all participating hospitals (Supplementary Table S1).

### 4.3. Study Design

The study was designed to be a multi-center, randomized, double-blind study with two arms. Patients who met the enrollment criteria and provided written informed consent were randomly assigned to 2 groups at a 1:1 ratio: the high dose (500 mg/day) group and the low dose (250 mg/day) group. Details of the randomization and blinding procedures are given below. Patients in either group received 500 mg/day or 250 mg/day PG2 injection 3 times per week for 4 weeks per cycle. It was intended that recruited patients would complete two cycles in this study. The study protocol is summarized in Figure 1A. Based on our previous studies and ethical considerations, the trial did not include a placebo control arm [14]. The study started in November 2012 and was completed in June 2017 (Clinical trial information: NCT01720550).

### 4.4. Patient Registration and Randomization

Patient registration/randomization was accepted from authorized investigators. All enrolled patients were randomly assigned to one of two study arms (High Dose Arm or Low Dose Arm). Each patient was randomized prior to commencement of the study, only after verification of all eligibility criteria on the day of screening. The block (of 4 subjects) randomization schedule was generated using a validated system that automates the random assignment of treatment arms to patient numbers. In this study, the sponsor supplied two dosage forms of PG2. However, it did not impact the randomization schedule because all patients were ensured to have the same dosage form throughout the study. Moreover, to balance the two treatment arms of patient numbers under different dosage forms, the remaining sterile powder was assigned to each site based on the block 4 as much as possible. This schedule linked sequential numbers to treatment codes allocated at random. After screening, enrolled patients were assigned to one of the above-mentioned two treatment arms according to the randomization schedule.

### 4.5. Blinding

As this was a randomized, double-blind study, the investigators, clinical research coordinator, and subjects remained blinded in terms of actual drug assignment during the period of study.

The investigators assigned an authorized person to prepare 500 or 250 mg PG2 in 500 mL normal saline in a separated and locked room. The authorized person was responsible for maintaining the blinding of the study.

The second measure to maintain blinding was to have the drug assignments for each subject sealed in individual blind-breaker envelopes and kept by the investigator during the period of the study. A full set of envelopes was also held by the sponsor. Envelopes were kept in secure locations with limited access in order to minimize the risk of inadvertent opening of the envelopes. Besides the investigators, study nurses, and subjects, all other personnel involved in the study (such as clinical monitor, statistician, and data management and data entry personnel) were also blinded in terms of study group assignments until data base lock.

### 4.6. Cancer-Related Fatigue Assessment

To evaluate the efficacy of the studied drug treatment, we used the Brief Fatigue Inventory-Taiwanese version to assess cancer-related fatigue before treatment (baseline) and at the end of each week of each cycle. The Brief Fatigue Inventory-Taiwanese version is a 9-item scale for measuring the severity of fatigue in cancer patients on a scale ranging from 0 to 10, with 0 indicating no fatigue and 10 indicating extreme fatigue. The 9 items include present, usual, and most severe episodes of fatigue over the last 24 h and interference with general activity, mood, walking ability, normal work, interpersonal relations, and enjoyment of life. The tabulated composite average score, as reported by the patient, represented the patient’s global fatigue severity score (Brief Fatigue Inventory-Taiwanese version score) [30,31]. A fatigue improvement responder was defined as a person with at least 10% improvement in the Brief Fatigue Inventory-Taiwanese version score compared to the baseline score at any efficacy evaluation point [19]. Fatigue improvement response rate was defined as the percentage of fatigue improvement responders in a group at every checkpoint. The primary goal of this study was to compare fatigue improvement response rates between the two study groups at the end of the first treatment cycle (at the end of the fourth week). Univariate and multivariate analyses were conducted to identify predictive factors for CRF in patients with PG2 treatment. KPS-related patient-grouping was based on a previous report [32]. In brief, cancer patients with KPS 50 or lower require nursing care with ambulatory <50% of time, while cancer patients with KPS 60 or higher require no assistance or occasional assistance with ambulatory >50% of time. Therefore, the threshold of KPS-related patient-grouping was set between 50 and 60.

### 4.7. Safety Evaluation

Adverse events were recorded as the onset of a new adverse effect or as an increase in the magnitude of existing adverse effects. Adverse events were tabulated and summarized using Medical Dictionary for Regulatory Activities (MedDRA version 9.1). Patient safety profiles included data on vital signs and results of physical examinations. Data on vital signs were recorded during screening and on infusion days. Physical examinations were conducted on the same days as the efficacy evaluation points.

### 4.8. Statistical Analysis

The study was set up to include at least 200 evaluable patients for efficacy analyses. Demographic characteristics, baseline clinical characteristics, quality of life status, and other laboratory values were summarized using descriptive statistics. For continuous variables, descriptive statistics, including the number of observations, mean, median, standard deviation, minimum, and maximum, were measured for each visit by each group. The differences with 95% confidence interval between groups were evaluated. For categorical variables, descriptive statistics, including frequency and percentages, were tabulated. The differences in proportions with 95% confidence interval between groups were evaluated for the efficacy endpoints.

This study compared the fatigue improvement response rate between the high dose and low dose arms by different cut-off values at the end of the first treatment cycle (i.e., the end of week 4) as the result of primary efficacy evaluation. For the week 4 primary endpoint, if the data were not available at time of the last visit of the first treatment cycle, the data from the first visit in the second treatment cycle were used to replace the data of the last visit of first treatment cycle for analyses. Each subject was classified as a responder or non-responder to fatigue improvement. Responders to fatigue improvement were determined by different cut-off values as the improvement in fatigue scores by at least 10% from baseline (the efficacy evaluation of the first visit in each treatment cycle).

For the univariate analysis, the two sample T-test or Wilcoxon rank-sum test was used to compare the difference between responders and non-responders for continuous variables; the Chi-square test or Fisher’s exact test was used to compare the difference between responders and non-responders for categorical variables. For multivariate analysis, the logistic regression model was used to compare the differences between responders and non-responders.

For drug-safety evaluation, the analyzed population included patients who were administered at least 1 dose of the study drug (safety dataset). The Chi-square or t-test was used to examine differences between the two groups at baseline and the end of each treatment cycle. A value of *p* < 0.05 was considered statistically significant.

## 5. Conclusions

The study demonstrated that PG2 injection is able to effectively reduce cancer-related fatigue in advanced cancer patients, and the efficacy of high and low doses of PG2 injection was statistically similar. Furthermore, patients with higher KPS may benefit more from treatment, and baseline KPS could be a promising predictor of PG2 efficacy. Based on these findings, the application of PG2 injection could be extended to cancer patients at earlier stages, as these patients generally have better performance status. However, further studies are required to verify the effect of PG2 injection in this patient population.

## Figures and Tables

**Table 1:** Patient information.

Variable/Statistics	High Dose Arm (*N* = 111)	Low Dose Arm (*N* = 103)
**Sex**		
Male	55 (49.55%)	66 (64.08%)
Female	56 (50.45%)	37 (35.92%)
**Age (years)**		
*n*	111	103
Mean (SD)	62.20 (10.69)	62.86 (11.50)
Median (min, max)	63 (28, 84)	64 (22, 91)
95% CI	(60.19, 64.21)	(60.62, 65.11)
**Body mass index (BMI) (kg/m^2^)**		
*n*	109	102
Mean (SD)	21.16 (4.30)	21.09 (3.99)
Median (min, max)	21.26 (12.18, 32.67)	20.81 (13.75, 37.01)
95% CI	(20.34, 21.98)	(20.30, 21.87)
**Baseline Karnofsky Performance Status (KPS) score (Cycle 1 Visit 1)**		
*n*	111	103
Mean (SD)	64.50 (14.82)	66.65 (14.06)
Median (min, max)	70 (30, 90)	70 (30, 90)
95% CI	(61.72, 67.29)	(63.90, 69.40)
**Baseline BFI score (Cycle 1 Visit 1)**		
*n*	111	103
Mean (SD)	6.80 (1.53)	6.76 (1.25)
Median (min, max)	6.6 (4, 10)	6.9 (4.1, 9.4)
95% CI	(6.51, 7.08)	(6.51, 7.00)

**Table 2:** Summary of Fatigue Improvement Response Rate.

Group/Cut-Off Points	High Dose Arm	Low Dose Arm	Differences among Groups with 95% CI
ITT Population	*N* = 111	*N* = 103	
10%	73 (65.77%)	67 (65.05%)	(−0.12, 0.13)
20%	57 (51.35%)	48 (46.60%)	(−0.09, 0.18)
30%	38 (34.23%)	36 (34.95%)	(−0.13, 0.12)
40%	21 (18.92%)	27 (26.21%)	(−0.18, 0.04)

**Table 3:** Multivariate analysis for responders and non-responders to Astragalus Polysaccharides (PG2) injection.

All Subjects					
	Cut-off Points = 10%			Multivariate Analysis	
Variable/Status	Responder (*N* = 140)	Non-Responder (*N* = 74)	Univariate Analysis *p*-value *	Odds Ratio (95% CI)	*p*-value **
**Age (years)**					
n	140	74	0.3085 ^W^	1.007 (0.978, 1.036)	0.6518
Mean (SD)	62.06 (11.28)	63.39 (10.66)			
Median (min, max)	62 (28, 91)	65 (22, 81)			
95% CI	(60.17, 63.94)	(60.92, 65.86)			
**Gender**					
Male	75 (53.57%)	46 (62.16%)	0.2279 ^C^	0.774 (0.387, 1.546)	0.4677
Female	65 (46.43%)	28 (37.84%)			
**Body mass index (BMI) (kg/m^2^)**					
<19	39 (28.26%)	27 (36.99%)	0.1935 ^C^	0.724 (0.364, 1.440)	0.3570
≥19	99 (71.74%)	46 (63.01%)			
number of missing	2	1			
**Body weight loss in previous 6 months**					
<5%	63 (45.65%)	30 (40.54%)	0.4746 ^C^	0.998 (0.512, 1.944)	0.9944
≥5%	75 (54.35%)	44 (59.46%)			
NA	2	0			
**Baseline KPS score**					
30–50	22 (15.71%)	31 (41.89%)	<0.0001 ^C^	0.253 (0.126, 0.504)	<0.0001
60–90	118 (84.29%)	43 (58.11%)			
**Baseline BFI score**					
4–6	72 (51.43%)	41 (55.41%)	0.5794 ^C^	0.885 (0.475, 1.647)	0.6998
7–10	68 (48.57%)	33 (44.59%)			
**Cancer Type: three categories**					
Lung cancer	22 (15.71%)	12 (16.22%)	0.2876 ^C^		
Breast cancer	22 (15.71%)	6 (8.11%)		1.297 (0.343, 4.905)	0.7020
other	96 (68.57%)	56 (75.68%)		0.957 (0.414, 2.208)	0.9173
**Albumin (g/dL)**					
<3.0	20 (14.29%)	11 (14.86%)	0.9088 ^C^	1.272 (0.518, 3.124)	0.5997
≥3.0	120 (85.71%)	63 (85.14%)			
**Hemoglobin (g/dL)**					
<10	48 (34.29%)	30 (40.54%)	0.3659 ^C^	0.767 (0.405, 1.452)	0.4148
≥10	92 (65.71%)	44 (59.46%)			
**Peripheral blood TLC (/µL)**					
<700	46 (32.86%)	18 (24.32%)	0.1947 ^C^	1.709 (0.846, 3.452)	0.1353
≥700	94 (67.14%)	56 (75.68%)			

* The Wilcoxon rank-sum test ^W^ was used to compare the difference between responders and non-responders for continuous variables; the Chi-squared test ^C^ was used to compare the difference between responders and non-responders for categorical variables. ** A logistic regression model was used to compare the differences between responders and non-responders.

**Table 4:** Summary of treatment-related adverse events with an incidence of greater than 2%.

System Organ Class/Preferred Term	High Dose Arm (*N* = 152)		Low Dose Arm (*N* = 155)	
	Event	Subject	Event	Subject
	E	*n* (%)	E	*n* (%)
Rash	24	14 (9.21%)	5	4 (2.58%)
Pyrexia	16	11 (7.24%)	5	5 (3.23%)
Feeling cold	9	8 (5.26%)	1	1 (0.65%)
Chills	7	4 (2.63%)	1	1 (0.65%)
Hypersensitivity	4	4 (2.63%)	0	0 (0.00%)

## Supplementary Materials

The following are available online at http://www.mdpi.com/2072-6694/11/2/128/s1, Figure S1: Summary of Fatigue Improvement Response Rate at Cycle 1 Week 4, Table S1: List of IRB approval information, Table S2: Cancer types of patients enrolled in this study, Table S3: Change in Brief Fatigue Inventory-Taiwanese version score at each evaluation time point, Table S4: Summary of adverse events, Table S5: Logistic regression analysis of fatigue improvement response rate for high dose arm subjects, Table S6: Logistic regression analysis of fatigue improvement response rate for low dose arm subjects.

## Abbreviation

BFI	Brief Fatigue Inventory-Taiwanese Form
BMI	Body mass index
C.I.	Confidence Interval
CNS	Central nervous system
CRF	Cancer-related fatigue
IRB	Institutional Review Board
ITT	Intention-to-treat
KPS	Karnofsky performance scale
MedDRA	Medical Dictionary for Regulatory Activities
SD	Standard deviation
TNF	Tumor necrosis factor

## References

[1] Mock V., Atkinson A., Barsevick A., Cella D., Cimprich B., Cleeland C., Donnelly J., Eisenberger M.A., Escalante C., Hinds P. (2000). NCCN Practice Guidelines for Cancer-Related Fatigue. Oncology (Williston Park).

[2] Vogelzang N.J., Breitbart W., Cella D., Curt G.A., Groopman J.E., Horning S.J., Itri L.M., Johnson D.H., Scherr S.L., Portenoy R.K. (1997). Patient, caregiver, and oncologist perceptions of cancer-related fatigue: Results of a tripart assessment survey. The Fatigue Coalition. Semin. Hematol..

[3] National Comprehensive Cancer Network WHO Definition Palliative Care, World Health Organization. NCCN Clinical Practice Guidelines in Oncology: Cancer Related Fatigue Version 1. https://s3.amazonaws.com/pfizerpro.com/fixtures/oncology/docs/NCCNFatigueGuidelines.pdf.

[4] Poulson M.J. (2001). Not just tired. J. Clin. Oncol..

[5] Andrykowski M.A., Curran S.L., Lightner R. (1998). Off-treatment fatigue in breast cancer survivors: A controlled comparison. J. Behav. Med..

[6] Jacobsen P.B., Hann D.M., Azzarello L.M., Horton J., Balducci L., Lyman G.H. (1999). Fatigue in women receiving adjuvant chemotherapy for breast cancer: Characteristics, course, and correlates. J. Pain Symptom Manag..

[7] Escalante C.P., Manzullo E.F., Lam T.P., Ensor J.E., Valdres R.U., Wang X.S. (2008). Fatigue and its risk factors in cancer patients who seek emergency care. J. Pain Symptom Manag..

[8] Bower J.E., Ganz P.A., Desmond K.A., Bernaards C., Rowland J.H., Meyerowitz B.E., Belin T.R. (2006). Fatigue in long-term breast carcinoma survivors: A longitudinal investigation. Cancer.

[9] Bruera E., Valero V., Driver L., Shen L., Willey J., Zhang T., Palmer J.L. (2006). Patient-controlled methylphenidate for cancer fatigue: A double-blind, randomized, placebo-controlled trial. J. Clin. Oncol..

[10] Hwang S.S., Chang V.T., Rue M., Kasimis B. (2003). Multidimensional independent predictors of cancer-related fatigue. J. Pain Symptom Manag..

[11] Minton O., Richardson A., Sharpe M., Hotopf M., Stone P. (2010). Drug therapy for the management of cancer-related fatigue. Cochrane Database Syst. Rev..

[12] Bower J.E. (2014). Cancer-related fatigue—Mechanisms, risk factors, and treatments. Nat. Rev. Clin. Oncol..

[13] Huang W.M., Liang Y.Q., Tang L.J., Ding Y., Wang X.H. (2013). Antioxidant and anti-inflammatory effects of Astragalus polysaccharide on EA.hy926 cells. Exp. Ther. Med..

[14] Chen H.W., Lin I.H., Chen Y.J., Chang K.H., Wu M.H., Su W.H., Huang G.C., Lai Y.L. (2012). A novel infusible botanically-derived drug, PG2, for cancer-related fatigue: A phase II double-blind, randomized placebo-controlled study. Clin. Investig. Med..

[15] Kuo Y.L., Chen C.H., Chuang T.H., Hua W.K., Lin W.J., Hsu W.H., Chang P.M., Hsu S.L., Huang T.H., Kao C.Y. (2015). Gene Expression Profiling and Pathway Network Analysis Predicts a Novel Antitumor Function for a Botanical-Derived Drug, PG2. Evid. Based Complement. Alternat. Med..

[16] McCulloch M., See C., Shu X.J., Broffman M., Kramer A., Fan W.Y., Gao J., Lieb W., Shieh K., Colford J.M. (2006). Astragalus-based Chinese herbs and platinum-based chemotherapy for advanced non-small-cell lung cancer: Meta-analysis of randomized trials. J. Clin. Oncol..

[17] Breitbart W., Alici Y. (2008). Pharmacologic treatment options for cancer-related fatigue: Current state of clinical research. Clin. J. Oncol. Nurs..

[18] Morrow G.R., Shelke A.R., Roscoe J.A., Hickok J.T., Mustian K. (2005). Management of cancer-related fatigue. Cancer Investig..

[19] Roth A.J., Nelson C., Rosenfeld B., Scher H., Slovin S., Morris M., O’Shea N., Arauz G., Breitbart W. (2010). Methylphenidate for fatigue in ambulatory men with prostate cancer. Cancer.

[20] Yeh E.T., Lau S.C., Su W.J., Tsai D.J., Tu Y.Y., Lai Y.L. (2011). An examination of cancer-related fatigue through proposed diagnostic criteria in a sample of cancer patients in Taiwan. BMC Cancer.

[21] Stasi R., Abriani L., Beccaglia P., Terzoli E., Amadori S. (2003). Cancer-related fatigue: Evolving concepts in evaluation and treatment. Cancer.

[22] Guo L., Bai S.P., Zhao L., Wang X.H. (2012). Astragalus polysaccharide injection integrated with vinorelbine and cisplatin for patients with advanced non-small cell lung cancer: Effects on quality of life and survival. Med. Oncol..

[23] Wang X.S., Zhao F., Fisch M.J., O’Mara A.M., Cella D., Mendoza T.R., Cleeland C.S. (2014). Prevalence and characteristics of moderate to severe fatigue: A multicenter study in cancer patients and survivors. Cancer.

[24] Karnofsky D.A.B., Burchenal J.H., MacLeod C.M. (1949). The Clinical Evaluation of Chemotherapeutic Agents in Cancer. Evaluation of Chemotherapeutic Agents.

[25] Bower J.E., Ganz P.A., Irwin M.R., Arevalo J.M., Cole S.W. (2011). Fatigue and gene expression in human leukocytes: Increased NF-kappaB and decreased glucocorticoid signaling in breast cancer survivors with persistent fatigue. Brain Behav. Immun..

[26] Ryan J.L., Carroll J.K., Ryan E.P., Mustian K.M., Fiscella K., Morrow G.R. (2007). Mechanisms of cancer-related fatigue. Oncologist.

[27] Clark J., Vagenas P., Panesar M., Cope A.P. (2005). What does tumour necrosis factor excess do to the immune system long term?. Ann. Rheum. Dis..

[28] Wang X., Li Y., Yang X., Yao J. (2013). Astragalus polysaccharide reduces inflammatory response by decreasing permeability of LPS-infected CaCO_2_ cells. Int. J. Biol. Macromol..

[29] Hou Y.C., Wu J.M., Wang M.Y., Wu M.H., Chen K.Y., Yeh S.L., Lin M.T. (2015). Modulatory Effects of Astragalus Polysaccharides on T-Cell Polarization in Mice with Polymicrobial Sepsis. Mediat. Inflamm..

[30] Lin C.C., Chang A.P., Chen M.L., Cleeland C.S., Mendoza T.R., Wang X.S. (2006). Validation of the Taiwanese version of the Brief Fatigue Inventory. J. Pain Symptom Manag..

[31] Mendoza T.R., Wang X.S., Cleeland C.S., Morrissey M., Johnson B.A., Wendt J.K., Huber S.L. (1999). The rapid assessment of fatigue severity in cancer patients: Use of the Brief Fatigue Inventory. Cancer.

[32] West H.J., Jin J.O. (2015). JAMA Oncology Patient Page. Performance Status in Patients With Cancer. JAMA Oncol..

