# Supplementary material for: Karnofsky Performance Status as A Predictive Factor for Cancer-Related Fatigue Treatment with Astragalus Polysaccharides (PG2) Injection—A Double Blind, Multi-Center, Randomized Phase IV Study
Source: Cancers (Basel). 2019 Jan 22;11(2):128. doi: 10.3390/cancers11020128 (PMC6406819; doi:10.3390/cancers11020128)
Supplement: Supplementary file 1 [file cancers-11-00128-s001.pdf]

*Supplementary Material*

# Karnofsky Performance Status as A Predictive Factor for Cancer-Related Fatigue Treatment with Astragalus Polysaccharides (PG2) Injection—A Double Blind, Multi-Center, Randomized Phase IV Study

**Cheng-Hsu Wang, Cheng-Yao Lin, Jen-Shi Chen, Ching-Liang Ho, Kun-Ming Rau, Jo-Ting Tsai, Cheng-Shyong Chang, Su-Peng Yeh, Chieh-Fang Cheng and Yuen-Liang Lai**

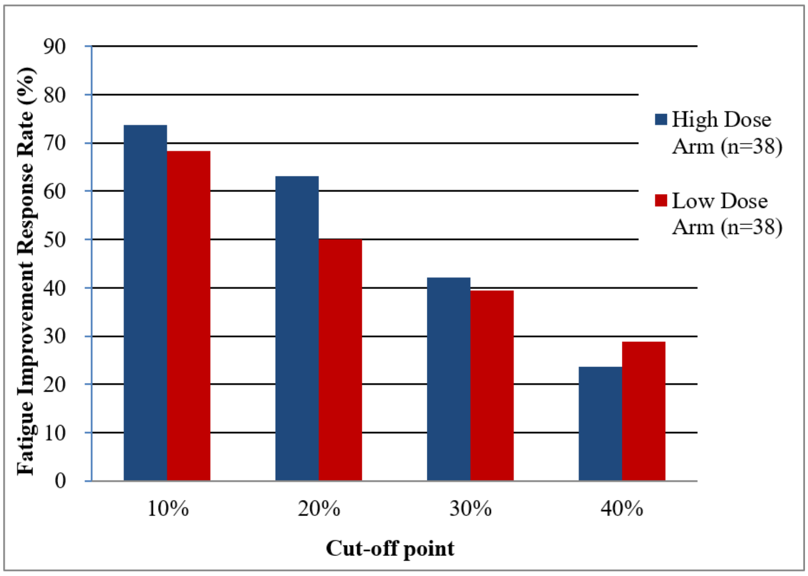


| **Co-variable Controlled ITT Population** | | | |
|---|---|---|---|
| | *N* = 38 | *N* = 38 | |
| 10% | 28 (73.68%) | 26 (68.42%) | (−0.15, 0.26) |
| 20% | 24 (63.16%) | 19 (50.00%) | (−0.09, 0.35) |
| 30% | 16 (42.11%) | 15 (39.47%) | (−0.19, 0.25) |
| 40% | 9 (23.68%) | 11 (28.95%) | (−0.25, 0.15) |

**Figure S1.** Summary of Fatigue Improvement Response Rate at Cycle 1 Week 4 (Patients without chemotherapy, radiation therapy, or steroid treatments).

**Table S1.** List of IRB approval information.

| IRB | Approval Dated | IRB No. |
|---|---|---|
| Joint Institutional Review Board | 2012/05/02 | 12-010-A |
| Institutional Review Board of Tri-Service General Hospital | 2012/04/18 | 2-101-01-001 |
| China Medical University & Hospital Research Ethics Committee | 2012/05/18 | DMR101-IRB2-032 |
| Taipei Medical University–Joint Institutional Review Board | 2012/07/03 | 201205017 |
| Institutional Review Board of the Chi Mei Medical Center | 2012/08/09 | 10108-L04 |
| Chang Gung Medical Foundation Institutional Review Board | 2012/11/02 | 101-2875A1 |
| Mackay Memorial Hospital Institutional Review Board | 2012/10/15 | 12CT026A |
| Institutional Review Board Changhua Christian Hospital | 2012/09/13 | 120803 |

**Table S2.** Cancer types of patients enrolled in this study.

| Variable/Statistics | High Dose Arm (*N* = 111) | Low Dose Arm (*N* = 103) |
|---|---|---|
| **Cancer type** | | |
| Transitional cell carcinoma | 1 (0.90%) | 0 (0.00%) |
| bile duct cancer | 4 (3.60%) | 5 (4.85%) |
| bladder cancer | 1 (0.90%) | 0 (0.00%) |
| breast cancer | 16 (14.41%) | 12 (11.65%) |
| colon cancer | 13 (11.71%) | 11 (10.68%) |
| duodenal caner | 1 (0.90%) | 0 (0.00%) |
| esophageal cancer | 6 (5.41%) | 8 (7.77%) |
| gallbladder cancer | 1 (0.90%) | 0 (0.00%) |
| gastric cancer | 10 (9.01%) | 10 (9.71%) |
| gastrointestinal stromal cancer | 0 (0.00%) | 1 (0.97%) |
| hepatobiliary cancer | 1 (0.90%) | 0 (0.00%) |
| hypopharyngeal cancer | 1 (0.90%) | 0 (0.00%) |
| laryngeal cancer | 0 (0.00%) | 2 (1.94%) |
| leukemia | 1 (0.90%) | 0 (0.00%) |
| lip and/or oral cavity cancer | 4 (3.60%) | 2 (1.94%) |
| liposarcoma | 2 (1.80%) | 2 (1.94%) |
| liver cancer | 4 (3.60%) | 6 (5.83%) |
| liver cancer, bile duct cancer | 0 (0.00%) | 1 (0.97%) |
| lung cancer | 20 (18.02%) | 14 (13.59%) |
| lymphoma | 1 (0.90%) | 2 (1.94%) |
| malignant peripheral nerve sheath tumor | 0 (0.00%) | 1 (0.97%) |
| nasopharyngeal cancer | 1 (0.90%) | 3 (2.91%) |
| oropharyngeal cancer | 0 (0.00%) | 1 (0.97%) |
| ovarian cancer | 2 (1.80%) | 3 (2.91%) |
| pancreatic cancer | 9 (8.11%) | 9 (8.74%) |
| pleural mesothelioma | 1 (0.90%) | 0 (0.00%) |
| Prostate cancer | 2 (1.80%) | 1 (0.97%) |
| rectal cancer | 2 (1.80%) | 3 (2.91%) |
| renal cancer | 1 (0.90%) | 1 (0.97%) |
| thymic cancer | 4 (3.60%) | 2 (1.94%) |
| tonsil cancer | 0 (0.00%) | 1 (0.97%) |
| ureteric cancer | 1 (0.90%) | 0 (0.00%) |
| uterine cancer | 0 (0.00%) | 1 (0.97%) |
| UNKNOWN | 1 (0.90%) | 1 (0.97%) |

**Table S3.** Change in Brief Fatigue Inventory-Taiwanese version score at each evaluation time point.

| Cycle/Visit | High Dose Arm (*N* = 111) | Low Dose Arm (*N* = 103) | Differences among Groups with 95% CI |
|---|---|---|---|
| **Cycle 1** | | | |
| Baseline | | | |
| n | 111 | 103 | |
| Mean (SD) | 6.80 (1.52) | 6.75 (1.25) | (−0.33, 0.43) |
| Median (min, max) | 6.55 (4, 10) | 6.88 (4.11, 9.44) | |
| 95% CI | (6.51, 7.08) | (6.50, 6.99) | |
| Week 1 Visit 3 | | | |
| n | 110 | 102 | |
| Mean (SD) | 6.05 (1.77) | 5.94 (1.58) | (−0.34, 0.57) |
| Median (min, max) | 6 (0, 9.33) | 5.88 (2.11, 9.55) | |
| 95% CI | (5.72, 6.39) | (5.63, 6.25) | |
| Week 1 Visit 3 Change from Baseline | | | |
| n | 110 | 102 | |
| Mean (SD) | −0.75 (1.25) | −0.80 (1.13) | (−0.28, 0.37) |
| Median (min, max) | −0.725 (−4.78, 2.89) | −0.78 (−4.34, 1.78) | |
| 95% CI | (−0.99, −0.52) | (−1.02, −0.58) | |
| * *p*-value | <0.0001 | <0.0001 | |
| Week 2 | | | |
| n | 108 | 102 | |
| Mean (SD) | 5.72 (1.89) | 5.49 (1.87) | (−0.29, 0.74) |
| Median (min, max) | 5.88 (0.55, 9.66) | 5.55 (1.22, 9.44) | |
| 95% CI | (5.36, 6.08) | (5.13, 5.86) | |
| Week 2 Change from Baseline | | | |
| n | 108 | 102 | |
| Mean (SD) | −1.12 (1.49) | −1.25 (1.71) | (−0.31, 0.57) |
| Median (min, max) | −1.11 (−5.22, 3.34) | −1.275 (−6.55, 3.23) | |
| 95% CI | (−1.40, −0.83) | (−1.58, −0.91) | |
| * *p*-value | <0.0001 | <0.0001 | |
| Week 3 | | | |
| n | 110 | 100 | |
| Mean (SD) | 5.58 (2.02) | 5.40 (1.85) | (−0.35, 0.71) |
| Median (min, max) | 5.605 (0, 10) | 5.33 (0.55, 9.44) | |
| 95% CI | (5.20, 5.97) | (5.03, 5.77) | |
| Week 3 Change from Baseline | | | |
| n | 110 | 100 | |
| Mean (SD) | −1.21 (1.80) | −1.37 (1.65) | (−0.31, 0.63) |
| Median (min, max) | −1 (−6.78, 3.89) | −1.28 (−7.22, 2.56) | |
| 95% CI | (−1.55, −0.87) | (−1.70, −1.04) | |
| * *p*-value | <0.0001 | <0.0001 | |
| Week 4 | | | |
| n | 109 | 103 | |
| Mean (SD) | 5.47 (1.95) | 5.29 (1.88) | (−0.34, 0.70) |
| Median (min, max) | 5.55 (0, 9.77) | 5.11 (0.33, 9.44) | |
| 95% CI | (5.10, 5.84) | (4.93, 5.66) | |
| Week 4 Change from Baseline | | | |
| n | 109 | 103 | |
| Mean (SD) | −1.32 (1.77) | −1.45 (1.83) | (−0.35, 0.62) |
| Median (min, max) | −1.44 (−6.23, 3.34) | −1.22 (−7.44, 3.11) | |
| 95% CI | (−1.65, −0.98) | (−1.81, −1.10) | |
| * *p*-value | <0.0001 | <0.0001 | |
| **Cycle 2** | | | |
| Week 1 Visit 3 | | | |
| n | 99 | 77 | |
| Mean (SD) | 5.11 (2.12) | 4.89 (2.02) | (−0.41, 0.84) |
| Median (min, max) | 5 (0, 9.77) | 4.77 (0, 9.66) | |

| | | | |
|---|---|---|---|
| 95% CI | (4.68, 5.53) | (4.43, 5.35) | |
| Week 1 Visit 3 Change from Baseline | | | |
| n | 99 | 77 | |
| Mean (SD) | −1.75 (1.81) | −1.83 (1.96) | (−0.48, 0.64) |
| Median (min, max) | −2 (−5.77, 2.44) | −1.78 (−7.88, 3.11) | |
| 95% CI | (−2.11, −1.39) | (−2.27, −1.38) | |
| * *p*-value | <0.0001 | <0.0001 | |
| Week 2 | | | |
| n | 89 | 71 | |
| Mean (SD) | 4.99 (2.22) | 5.12 (1.97) | (−0.79, 0.54) |
| Median (min, max) | 4.66 (0.22, 9.66) | 4.66 (1, 9.66) | |
| 95% CI | (4.52, 5.46) | (4.65, 5.58) | |
| Week 2 Change from Baseline | | | |
| n | 89 | 71 | |
| Mean (SD) | −1.80 (2.16) | −1.60 (1.92) | (−0.85, 0.45) |
| Median (min, max) | −2.11 (−6, 3.56) | −1.77 (−7.33, 3.22) | |
| 95% CI | (−2.26, −1.35) | (−2.06, −1.15) | |
| * *p*-value | <0.0001 | <0.0001 | |
| Week 3 | | | |
| n | 81 | 67 | |
| Mean (SD) | 4.82 (2.08) | 4.70 (2.05) | (−0.56, 0.79) |
| Median (min, max) | 4.55 (0, 9.88) | 4.22 (0.33, 8.77) | |
| 95% CI | (4.36, 5.28) | (4.20, 5.20) | |
| Week 3 Change from Baseline | | | |
| n | 81 | 67 | |
| Mean (SD) | −2.00 (2.15) | −1.96 (2.06) | (−0.73, 0.65) |
| Median (min, max) | −1.89 (−6.78, 3.78) | −2 (−6.55, 3.22) | |
| 95% CI | (−2.48, −1.53) | (−2.46, −1.46) | |
| * *p*-value | <0.0001 | <0.0001 | |
| Week 4 | | | |
| n | 75 | 65 | |
| Mean (SD) | 4.76 (2.24) | 4.54 (2.14) | (−0.52, 0.95) |
| Median (min, max) | 4.44 (0.11, 10) | 4.33 (0, 9.11) | |
| 95% CI | (4.24, 5.28) | (4.01, 5.07) | |
| Week 4 Change from Baseline | | | |
| n | 75 | 65 | |
| Mean (SD) | −2.06 (2.07) | −2.09 (2.23) | (−0.69, 0.74) |
| Median (min, max) | −2.22 (−7.23, 3.45) | −2.11 (−7.55, 3.22) | |
| 95% CI | (−2.54, −1.59) | (−2.64, −1.54) | |
| * *p*-value | <0.0001 | <0.0001 | |
| [Cycle 2- Week 4 change from baseline]-[Cycle 1- Week 4 change from baseline] | | | |
| n | 75 | 65 | |
| Mean (SD) | −0.56 (1.56) | −0.37 (1.66) | (−0.72, 0.35) |
| Median (min, max) | −0.55 (−5.44, 3.56) | −0.11 (−5.22, 3.89) | |
| 95% CI | (−0.91, −0.20) | (−0.78, 0.04) | |
| * *p*-value | 0.0028 | 0.0768 | |

* Compared to baseline.

**Table S4.** Summary of adverse events.

| Status/Category | High Dose Arm (*N* = 152) | | Low Dose Arm (*N* = 155) | |
|---|---|---|---|---|
| | Event | Subject | Event | Subject |
| | E | n (%) | E | n (%) |
| Total number of adverse events | | | | |
| - | 872 (100%) | 141 (92.76%) | 878 (100.00%) | 148 (95.48%) |
| Treatment-related adverse events | | | | |
| - | 76 (8.72%) | 33 (21.71%) | 57 (6.49%) | 21 (13.55%) |
| Total number of severe adverse events | | | | |
| - | 64 (7.34%) | 53 (34.87%) | 81 (9.23%) | 72 (46.45%) |
| Severity | | | | |
| Grade 1 Mild | 442 (50.69%) | 112 (73.68%) | 391 (44.53%) | 116 (74.84%) |
| Grade 2 Moderate | 302 (34.63%) | 100 (65.79%) | 290 (33.03%) | 93 (60.00%) |
| Grade 3 Severe | 86 (9.86%) | 48 (31.58%) | 117 (13.33%) | 57 (36.77%) |
| Grade 4 Life-threatening or disabling | 19 (2.18%) | 14 (9.21%) | 43 (4.90%) | 18 (11.61%) |
| Grade 5 Death | 23 (2.64%) | 22 (14.47%) | 37 (4.21%) | 37 (23.87%) |
| Relationship to Study Drug | | | | |
| Unrelated | 729 (83.60%) | 129 (84.87%) | 769 (87.59%) | 140 (90.32%) |
| Unlikely | 67 (7.68%) | 26 (17.11%) | 52 (5.92%) | 19 (12.26%) |
| Possibly | 33 (3.78%) | 17 (11.18%) | 47 (5.35%) | 14 (9.03%) |
| Probably | 24 (2.75%) | 9 (5.92%) | 5 (0.57%) | 4 (2.58%) |
| Definitely | 19 (2.18%) | 8 (5.26%) | 5 (0.57%) | 3 (1.94%) |
| Outcome | | | | |
| Recovered | 417 (47.82%) | 109 (71.71%) | 377 (42.94%) | 110 (70.97%) |
| Recovered with residual effects | 3 (0.34%) | 3 (1.97%) | 7 (0.80%) | 5 (3.23%) |
| Continuing | 314 (36.01%) | 98 (64.47%) | 304 (34.62%) | |
| Death | 123 (14.11%) | 23 (15.13%) | 181 (20.62%) | 37 (23.87%) |
| Lost to follow-up | 15 (1.72%) | 1 (0.66%) | 9 (1.03%) | 1 (0.65%) |
| Action Taken with Study Drug | | | | |
| No action | 353 (40.48%) | 111 (73.03%) | 320 (36.45%) | 106 (68.39%) |
| Treatment given | 500 (57.34%) | 124 (81.58%) | 527 (60.02%) | 130 (83.87%) |
| Withdrawn from study | 19 (2.18%) | 19 (12.50%) | 31 (3.53%) | 29 (18.71%) |

**Table S5.** Logistic regression analysis of fatigue improvement response rate for high dose arm subjects.

| **Variable/Status** | **High Dose Arm** | | | | |
|---|---|---|---|---|---|
| | **Cut-off Points = 10%** | | | **Multivariate Analysis** | |
| | **Responder (*N* = 73)** | **Non-Responder (*N* = 38)** | **Univariate Analysis *p*-Value *** | **Odds Ratio (95% CI)** | ***p*-Value **** |
| Age (years) | | | | | |
| n | 73 | 38 | 0.1018 [T] | 0.979 (0.931, 1.029) | 0.4074 |
| Mean (SD) | 61.00 (10.90) | 64.50 (10.00) | | | |
| Median (min, max) | 62 (28, 84) | 65 (40, 81) | | | |
| 95% CI | (58.46, 63.54) | (61.21, 67.79) | | | |
| Gender | | | | | |
| Male | 35 (47.95 %) | 20 (52.63 %) | 0.6394 [C] | 1.088 (0.403, 2.933) | 0.8683 |
| Female | 38 (52.05 %) | 18 (47.37 %) | | | |
| Body mass index (BMI) (kg/m$^2$) | | | | | |
| <19 | 20 (28.17 %) | 13 (34.21 %) | 0.5130 [C] | 0.760 (0.263, 2.198) | 0.6126 |
| ≥19 | 51 (71.83 %) | 25 (65.79%) | | | |
| number of missing | 2 | 0 | | | |
| Body weight loss in previous 6 months | | | | | |
| <5% | 26 (36.62 %) | 17 (44.74%) | 0.4086 [C] | 0.601 (0.215, 1.680) | 0.3319 |
| ≥5% | 45 (63.38%) | 21 (55.26 %) | | | |
| NA | 2 | 0 | | | |
| Baseline KPS score | | | | | |
| 30–50 | 11 (15.07 %) | 19 (50.00 %) | <0.0001 [C] | 0.138 (0.048, 0.400) | 0.0003 |
| 60–90 | 62 (84.93 %) | 19 (50.00 %) | | | |
| Baseline BFI score | | | | | |
| 4–6 | 39 (53.42 %) | 22 (57.89 %) | 0.6533 [C] | 0.662 (0.255, 1.716) | 0.3959 |
| 7–10 | 34 (46.58 %) | 16 (42.11 %) | | | |
| Cancer Type: three category | | | | | |
| Lung cancer | 13 (17.81 %) | 7 (18.42 %) | 0.6994 [C] | | |
| Breast cancer | 12(16.44 %) | 4 (10.53 %) | | 0.944 (0.155, 5.743) | 0.9504 |
| other | 48 (65.75 %) | 27 (71.05 %) | | 0.960 (0.288, 3.200) | 0.9464 |
| Albumin (g/dL) | | | | | |
| <3.0 | 10 (13.70 %) | 5 (13.16 %) | 0.9370 [C] | 1.799 (0.426, 7.602) | 0.4245 |
| ≥3.0 | 63 (86.30 %) | 33 (86.84 %) | | | |
| Hemoglobin (g/dL) | | | | | |
| <10 | 27 (36.99 %) | 17 (44.74 %) | 0.4283 [C] | 0.508 (0.195, 1.322) | 0.1651 |

| | | | | | |
|---|---|---|---|---|---|
| ≥10 | 46 (63.01 %) | 21 (55.26 %) | | | |
| Peripheral blood TLC (/uL) | | | | | |
| <700 | 24 (32.88 %) | 10 (26.32 %) | 0.4768 C | 1.624 (0.556, 4.743) | 0.3751 |
| ≥700 | 49 (67.12 %) | 28 (73.68 %) | | | |

* The two sample *t*-test T was used to compare the difference between responders and non-responders for continuous variables; the Chi-squared test C was used to compare the difference between responders and non-responders for categorical variables. ** A logistic regression model was used to compare the differences between responders and non-responders.

**Table S6.** Logistic regression analysis of fatigue improvement response rate for low dose arm subjects.

| | **Low Dose Arm** | | | | |
|---|---|---|---|---|---|
| | **Cut-off Points = 10%** | | | **Multivariate Analysis** | |
| **Variable/Status** | **Responder (*N* = 67)** | **Non-responder (*N* = 36)** | **Univariate Analysis *p*-Value *** | **Odds Ratio (95% CI)** | ***p*-Value **** |
| Age (years) | | | | | |
| n | 67 | 36 | 0.8735 W | 1.019 (0.980, 1.060) | 0.3457 |
| Mean (SD) | 63.21 (11.65) | 62.22 (11.34) | | | |
| Median (min, max) | 64 (28, 91) | 65 (22, 76) | | | |
| 95% CI | (60.37, 66.05) | (58.38, 66.06) | | | |
| Gender | | | | | |
| Male | 40 (59.70 %) | 26 (72.22 %) | 0.2066 C | 0.676 (0.224, 2.036) | 0.4864 |
| Female | 27 (40.30 %) | 10 (27.78 %) | | | |
| Body mass index (BMI) (kg/m$^2$) | | | | | |
| <19 | 19 (28.36 %) | 14 (40.00 %) | 0.2328 C | 0.700 (0.250, 1.958) | 0.4965 |
| ≥19 | 48 (71.64 %) | 21 (60.00%) | | | |
| number of missing | 0 | 1 | | | |
| Body weight loss in previous 6 months | | | | | |
| <5% | 37 (55.22 %) | 13 (36.11 %) | 0.0642 C | 1.709 (0.661, 4.421) | 0.2691 |
| ≥5% | 30 (44.78%) | 23 (63.89 %) | | | |
| Baseline KPS score | | | | | |
| 30–50 | 11 (16.42 %) | 12 (33.33 %) | 0.0494 C | 0.335 (0.117, 0.960) | 0.0417 |
| 60–90 | 56 (83.58 %) | 24 (66.67 %) | | | |
| Baseline BFI score | | | | | |
| 4–6 | 33 (49.25 %) | 19 (52.78 %) | 0.7330 C | 0.932 (0.381, 2.280) | 0.8767 |
| 7–10 | 34 (50.75 %) | 17 (47.22 %) | | | |
| Cancer Type: three category | | | | | |
| Lung cancer | 9 (13.43 %) | 5 (13.89 %) | 0.4040 F | | |

| | | | | | |
|---|---|---|---|---|---|
| Breast cancer | 10 (14.93 %) | 2 (5.56 %) | | 1.948 (0.214, 17.735) | 0.5541 |
| other | 48 (71.64 %) | 29 (80.56 %) | | 1.045 (0.286, 3.819) | 0.9468 |
| Albumin (g/dL) | | | | | |
| <3.0 | 10 (14.93 %) | 6 (16.67 %) | 0.8161 [C] | 0.841 (0.230, 3.076) | 0.7934 |
| ≥3.0 | 57 (85.07 %) | 30 (83.33 %) | | | |
| Hemoglobin (g/dL) | | | | | |
| <10 | 21 (31.34 %) | 13 (36.11 %) | 0.6237 [C] | 1.286 (0.467, 3.539) | 0.6266 |
| ≥10 | 46 (68.66 %) | 23 (63.89 %) | | | |
| Peripheral blood TLC (/uL) | | | | | |
| <700 | 22 (32.84 %) | 8 (22.22 %) | 0.2583 [C] | 1.866 (0.656, 5.304) | 0.2419 |
| ≥700 | 45 (67.16 %) | 28 (77.78 %) | | | |

* The Wilcoxon rank-sum test [W] was used to compare the difference between responders and non-responders for continuous variables; the Chi-squared test [C] or Fisher's exact test [F] was used to compare the difference between responders and non-responders for categorical variables. ** A logistic regression model was used to compare the differences between responders and non-responders.

© 2019 by the authors. Licensee MDPI, Basel, Switzerland. This article is an open access article distributed under the terms and conditions of the Creative Commons Attribution (CC BY) license (http://creativecommons.org/licenses/by/4.0/).
